# Yeast–Yeast Interactions: Mechanisms, Methodologies and Impact on Composition

**DOI:** 10.3390/microorganisms8040600

**Published:** 2020-04-20

**Authors:** Fanny Bordet, Alexis Joran, Géraldine Klein, Chloé Roullier-Gall, Hervé Alexandre

**Affiliations:** 1 Univ. Bourgogne Franche-Comté, AgroSup Dijon, PAM UMR A 02.102, F-21000 Dijon, France-IUVV Equipe VAlMiS, rue Claude Ladrey, BP 27877, 21078 Dijon CEDEX, France; 2 Lallemand SAS, 19, rue des Briquetiers, BP 59, 31702 Blagnac CEDEX, France

**Keywords:** yeast–yeast interactions, wine, mixed culture, methodologies, fermentation conditions, omics, sensory

## Abstract

During the winemaking process, alcoholic fermentation is carried out by a consortium of yeasts in which interactions occurs. The consequences of these interactions on the wine matrix have been widely described for several years with the aim of controlling the winemaking process as well as possible. In this review, we highlight the wide diversity of methodologies used to study these interactions, and their underlying mechanisms and consequences on the final wine composition and characteristics. The wide variety of matrix parameters, yeast couples, and culture conditions have led to contradictions between the results of the different studies considered. More recent aspects of modifications in the composition of the matrix are addressed through different approaches that have not been synthesized recently. Non-volatile and volatile metabolomics, as well as sensory analysis approaches are developed in this paper. The description of the matrix composition modification does not appear sufficient to explain interaction mechanisms, making it vital to take an integrated approach to draw definite conclusions on them.

## 1. Introduction

The transformation of grape must into wine is a complex process involving various microorganisms: yeasts, molds, and bacteria. The main step, alcoholic fermentation, is performed by yeasts. In natural fermentation, microflora comes from grape berries but also from winery equipment and surroundings. Yeast biodiversity on grape berries is governed by various biotic and abiotic factors such as grape variety, climatic conditions and viticultural practices [1,2,3]. Yeasts present on grapes are mainly from non-*Saccharomyces* genera (essentially *Hanseniaspora*, *Candida*, *Kluyveromyces*, *Metschnikowia*, *Pichia*, *Cryptococcus* and *Rhodotorula* [3]) while *Saccharomyces* genera are very rare. However, although non-*Saccharomyces* yeasts initiate fermentation and develop during the first hours, their population declines rapidly in favor of *Saccharomyces cerevisiae* (*S. cerevisiae*), which becomes the dominant species until the end of alcoholic fermentation. The evolution of yeast populations during fermentation seems to be linked to several modifications that make the medium more selective. The establishment of nutrient depletion, anaerobic conditions, increased acidity, the production of sulfur dioxide, and increasing levels of ethanol (up to 15%v/v) results in a drop in yeast diversity [2]. This modification of the matrix environment allows the survival of *S. cerevisiae* because of its overall better resistance to stress compared to non-*Saccharomyces* species [4].

Producers have used wine starters for many decades to ensure proper fermentation initiation and the quality and reproducibility of wine. Indeed, starter yeasts allow efficient fermentation management that limits contaminations and avoids deviations due to interrupted or sluggish fermentations [5]. These starter yeasts are selected for their specific metabolic properties: resistance to various stresses, fermentation capacity, or the presence of enzymatic activities [6]. The ability of *S. cerevisiae* to grow in a selective medium as described above, to carry out efficient and quick alcoholic fermentations, make this species a tool of choice as an oenological starter [5].

However, in recent years, non-*Saccharomyces* yeasts have been used for wine production since several yeast species have shown high oenological potential [7,8]. Indeed, yeasts like *Saccharomyces* non-*cerevisiae* [9,10], non-*Saccharomyces* [6,7,11,12,13,14,15,16], and even natural hybrids [17,18,19,20,21] are of interest, because their different metabolisms compared to *S. cerevisiae* brings diversity to quantitative and qualitative composition of final wine (for example, ethanol content, organic acids, aroma production) [3,22,23]. Nevertheless, all these studies show that the utilization of these yeasts, in combination with *S. cerevisiae*, as wine starters, is still a challenge, since the results are unpredictable and lack of reproducibility. The conduct of fermentations by managing the simultaneous or successive implantation of different strains to obtain the desired impacts on wine has not yet been mastered. It is therefore necessary to understand the phenomena involved in the evolution of the yeast ecosystem during alcoholic fermentation, to control these mixed cocultures more efficiently. In recent years, numerous researchers have furthered research in understanding interaction mechanisms between microorganisms, since these interactions impact not only the population dynamics but also the metabolism of each strain, with consequences on the compounds produced, and eventually on final wine quality.

Authors have shown the existence of different interaction mechanisms between yeasts: competition for nutrients, the production of inhibitory or toxic compounds, the modification of metabolism by a quorum-sensing answer or induced by cell-contact. But all these results highlight that yeast interactions during wine alcoholic fermentation are very complex because of the variations according to yeasts (species, strain), medium composition, and abiotic conditions (oxygen, temperature). These fickle results (as described in a recent review from Conacher et al. (2019) [24]) seem to be linked to the sheer diversity of the methodologies employed. Each team often works with different strains including commercial and indigenous strains, different types of culture media, various matrixes (synthetic must or musts from different grape varieties) and also different culture modalities Each of these factors can impact the population and fermentation dynamics, by tipping the balance of a fragile equilibrium between species one way or another. Despite the great need for an overview of these methodological differences, so far none is available, although it would lead to better comparisons of results, and provide a synthesis of standard protocols for newcomers in the field.

The objective of this review is to highlight recent scientific developments concerning yeast–yeast interactions. First, the different methodologies employed will be discussed, including recent contributions from transcriptomic and metabolomic approaches. The impact of interactions on volatile and non-volatile composition will then be considered. Finally, the consequences of interactions on wine sensory characteristics will be discussed in-depth.

## 2. Methodologies

### 2.1. Parameters: Inoculation and Culture Conditions

Numerous studies have been performed on mixed cultures between *S. cerevisiae* and non-*Saccharomyces* to understand how yeast interactions can impact wine quality. Authors have monitored population dynamics, fermentation parameters, metabolite production, especially aroma compound production, and highlighted interaction mechanisms. But contradictory results can be found in this field, as shown in Table 1 and Table S1, which include the conditions and results of experiments for several couples of yeasts, those most studied to improve wine quality.

Variability in population dynamics results can be observed depending on the various studies. The *S. cerevisiae* population is not affected in most experiments by the presence of another yeast, even if some exceptions exist [12,29,39,46,47]. On the other hand, the presence of *S. cerevisiae* usually negatively impacts non-*Saccharomyces* growth and early decline and even early death are often observed, but some authors have observed the stability of non-*Saccharomyces* yeasts during a longer period [33,45]. Fermentation kinetics can also be different. Mixed cultures with non*-Saccharomyces* yeasts can lead either to complete fermentations (within different timeframes) [48,49], or to incomplete fermentation [12,33]. The production of metabolites such as glycerol, acids, and aroma compounds is also variable [31,33].

Yeasts are often inoculated at a cell count of 10^6^ cells/mL since this corresponds to the conditions occurring in natural fermentation [50], in which there is dominance of non-*Saccharomyces* populations at the early stage, but inoculation density can vary between 5.10^4^ [26] and 2.10^7^ cells/mL [29,51].

The first hypothesis to explain this diversity of results is medium composition, which is known to impact yeast physiology, metabolism, and yeast interactions. Table 1 and Table S1 show that numerous authors choose to use real grape juice or must to approach winemaking conditions. But natural grape must is not standardized and its composition varies depending, for example, on the year, harvest time, and grape variety. Englezos et al. (2016) [12] and Nisiotou et al. (2018) [49] both conducted mixed fermentation with *Starmerella bacillaris* (*S. bacillaris*) in similar conditions (temperature and inoculation) but obtained different results. Indeed, contradictory results are reported in terms of non-*Saccharomyces* persistence and fermentation completion reflecting the influence of the matrix composition on yeast interactions. However, other differences (must sterilization, yeast strain) in methodology can also explain these discrepancies.

Initial sugar concentration can impact yeast growth but also the capacity of yeasts to interact with other yeasts. The ability to take up glucose varies with glucose concentration with a species-dependent effect. Outside the 160-190 g/L range, non-*Saccharomyces* yeasts are less able to take up glucose and become less able to compete with *S. cerevisiae* in mixed cultures [27]. In addition, initial sugar concentration and the amount of sugar metabolized by *S. cerevisiae* could have an impact on toxic compound production and further on population dynamics in mixed cultures: a delay in *Hanseniaspora guillermondii* (*H. guillermondii)* death can be observed when the initial sugar concentration is 100 g/L, compared to a standard medium with 200 g/L [52]. These effects are not always verified since *Lachancea thermotolerans (L. thermotolerans)* can survive until the end of fermentation with 200 g/L of initial sugars [39] but not with 160 g/L of initial sugars [31], although other factors can be involved (total population, oxygenation, strain). At high sugar concentrations (up to 200-300 g/L), yeasts can delay their growth and have a lower growth rate, with a possible effect on population dynamics [53]. However, *L. thermotolerans* can be used in the mixed fermentation of sun-dried grapes with a very high sugar content and become the dominant strain if inoculated at a high ratio [45].

Other medium components are also important to manage since *S. cerevisiae* and non-*Saccharomyces* yeasts have different needs and do not metabolize nutrients in the same way. A key point is nitrogen source quality since ammonium and amino acids may be assimilated or not, with various rates according to the strain and culture conditions like temperature [54,55,56,57]. In addition, competition for nutrients can occur between strains and limiting nutrients concentrations can increase their interactions. For example, in sequential mixed cultures, an insufficient initial amount of assimilable nitrogen can be entirely consumed by non-*Saccharomyces* yeast before the inoculation of *S. cerevisiae*, resulting in incomplete fermentation [51]. Moreover, nitrogen availability has an impact on the ability of yeast to compete with other strains. When nitrogen is limited, indigenous *S. cerevisiae* strains are more competitive toward commercial *S. cerevisiae* and can co-dominate fermentation; they have higher nitrogen demand and can quickly remove nitrogen from the medium, which is then no longer available for commercial strains [58]. In addition, as the production of aromatic alcohols (as tyrosol, tryptophol), known as quorum sensing molecules, is linked to nitrogen metabolism, the amount of nitrogen can impact this production or the effects of these molecules [59].

All these differences in must composition (sugars, YAN) can cause variabilities in yeast interactions and complicate understanding of the mechanisms involved. Considering this must variability, some authors choose to standardize their fermentation medium by supplementing must with sugar or nitrogen sources. However, referring to Table 1 and Table S1, it can be seen that the target level is not always the same, making comparisons between studies difficult. Moreover, Englezos et al. (2018) [60] observed that even with standardized sugar and YAN composition, grape variety still has a significant impact on non-*Saccharomyces* persistence during the fermentation process, hinting at the impossibility of effectively standardizing real must.

An alternative to these limits would be to use a synthetic medium with a fixed composition that can simulate natural must. Some researchers have chosen to use a quite simple medium such as a classical laboratory medium supplemented with sugars [26,28], while others have used compositions more similar to natural must, called “synthetic must” or “synthetic juice” [61]. Here, several key choices subsist, as some nutrients can impact yeast dynamics: the proportion of fructose in sugars [62,63], vitamins, and growth factors [64]. Authors have usually used this type of media to allow for effective standardization while studying the impact of a specific factor on interactions. For example, Wang et al. in 2016 [65] used synthetic must to show strain dependence in interactions between multiple *Saccharomyces/* non*-Saccharomyces* couples. Shekhawat et al. (2017) [29] also used synthetic grape juice to study the impact of must oxygenation on mixed cultures of *L. thermotolerans/S. cerevisiae*.

The inoculation procedure is also an important parameter. Authors have conducted mixed fermentations with the simultaneous or sequential inoculation of yeasts, with various times between both inoculations. The addition of *S. cerevisiae* after non-*Saccharomyces* can delay their death and increase their influence on wine characteristics. *L. thermotolerans* can be present until the end of fermentation, when *S. cerevisiae* is added 24, 48, or 72 h afterwards [33,39], but its population decreases drastically when a longer delay is used (4 days) [35,41]. However, some authors have observed a decrease with inoculation performed after 24 h [46], 48 h [14,31,44], or 72 h [37,66] indicating that other factors (medium composition, temperature, oxygenation) can interact. Non-*Saccharomyces* can also, in sequential culture, become the dominant strain and negatively impact *S. cerevisiae* growth, as was shown for *L. thermotolerans* [39], and *S. bacillaris* [12,47,49,60,67,68,69].

Different inoculation modes can also be coupled with different inoculation ratios between the two strains present in mixed culture, which has great importance for population dynamics. In spontaneous fermentation conditions, the yeast population of freshly extracted must is overwhelmingly constituted by non-*Saccharomyces*, with *S. cerevisiae* accounting for less than 1% of the total yeast population [70]. To simulate natural conditions, some researchers have inoculated using a large amount of non-*Saccharomyces* compared to *S. cerevisiae*, with the objective of improving non-*Saccharomyces* persistence during mixed fermentation. Usually, a ratio favoring a specific yeast has a positive impact on the latter’s population dynamics; longer persistence, higher population, and dominance, as was shown for *S. bacillaris* [32], *Saccharomyces kudriavzevii (S. kudriavzevii)* [9], *H. guilliermondii* [52], *L. thermotolerans* [45]. However, adjusting inoculation ratios is not always enough to obtain the persistence of non-*Saccharomyces* yeasts [48,51]. The initial amount of yeasts can also have an impact since the death of non-*Saccharomyces* yeasts can be linked to the presence of the high cell density of *S. cerevisiae* (*H. guilliermondii* declines when *S. cerevisiae* reaches 10^7^ CFU/mL [52]).

The physiological state of yeast can also have an impact on interaction. Branco et al. (2017) [71] showed that *S. cerevisiae* induces the death of non-*Saccharomyces* yeasts in mixed cultures, by different mechanisms, depending on its physiological state when mixed culture begins: cell-contact is involved when *S. cerevisiae* is in stationary phase and not when it is in mid-exponential phase. A potential explanation could be the accumulation of antimicrobial peptides on the cell-surface during *S. cerevisiae* growth, according to these authors.

Other culture parameters such as temperature can also impact population dynamics and ecosystems, since yeasts have different optimal growth temperatures [72]. *S. cerevisiae* is better adapted to higher temperature and even modifies temperature through heat production during fermentation [73]. The application of low temperature can favor the growth, survival, and even dominance of non-*cerevisiae* species [74,75,76] and of non-*Saccharomyces* yeasts [77]. Temperature can also increase the competitive ability of non-*Saccharomyces*: *L. thermotolerans* has an inhibitory effect on *S. cerevisiae* growth at 20 °C, while at 30 °C, *S. cerevisiae* competes better and *L. thermotolerans* biomass declines after 4 days [33]. Maturano et al. (2016) [78] even showed that the temperature of cold maceration prior to alcoholic fermentation can positively impact interspecific distribution. Temperature can also impact yeast metabolism. The response of *S. cerevisiae* against the presence of another strain (coculture), by gene expression, is indeed dependent on temperature (transcriptional response higher at 12 °C than at 20 °C) [10]. The variation of fermentation temperature may be involved in the variability of the results obtained by various authors. Differences in fermentation kinetics observed with red and white must (complete at 17 and 24 days respectively) by Whitener et al. and Becker Whitener et al. can be partly explained by differences of temperature (25 and 15 °C) [36,41]. Englezos et al. also explained the different impacts on ethanol content in their works of 2016 and 2018 by temperature differences between protocols [79]. Bagheri et al. recently showed that the population dynamics in a multi-species yeast consortium were affected by temperature, influencing consequently aroma compounds production [80].

One other key point used to explain the differences observed in population dynamics is oxygen availability, induced by different conditions of oxygenation and agitation in various authors’ protocols. Oxygen is indeed also known to have impacts on yeast interactions. Non-*Saccharomyces* yeasts are usually less tolerant to low oxygen availability than *S. cerevisiae* [30]: oxygen can increase their survival in mixed culture without affecting *S. cerevisiae*, resulting in a species-dependent variation in population dynamics (persistence) [29,43,67].

On the contrary, removing oxygenation and agitation altogether and allowing fermentations to occur in static conditions makes it possible to get closer to standard vinification conditions. These conditions, besides limiting oxygen intake, also allow the natural sedimentation of yeasts to occur. This sedimentation leads to heterogenous cell distribution, with an increase of local cell density in the sediment and a decrease in the supernatant. Cell density, as shown by Nissen et al. (2003) [26] is a key factor in yeast interactions. Cell density can also favor cell–cell contacts and coaggregation mechanisms which both seem to be involved in the population dynamics and metabolic changes observed in mixed cultures [24].

Studies have also shown that yeast interactions are heavily strain-dependent. Perrone et al. (2013) [81] studied 99 strains of autochthonous *S. cerevisiae* in must and showed that their dominance behavior varies and is expressed only when *S. cerevisiae* senses other yeasts in the same environment. As far as interactions between non-*Saccharomyces* and *S. cerevisiae* are concerned, mechanisms can be influenced by the strain chosen for both species. Wang et al. (2016) [65] observed that culturability loss of non-*Saccharomyces* because of interactions with *S. cerevisiae* is species- and strain-dependent. On the other hand, Englezos et al. (2016, 2019) [12,69] showed that *S. cerevisiae* strain choice also has a key impact on population dynamics *(S. bacillaris* is more or less able to dominate various *S. cerevisiae* strains), sugar consumption, wine composition (ethanol, glycerol, acid production), wine volatile compounds (decrease or increase of aroma production depending on the *S. cerevisiae* strain).

### 2.2. Yeast Interactions: Understanding Population Dynamics

When yeasts are cultivated together in the same medium, as happens in natural must, different interactions occur, with visible and measurable impacts on population dynamics (dominance of one strain, decline or death of others) and cell physiology.

Researchers usually approach population monitoring quantitatively: using the methods described below, they manage to get an overview of general population dynamics. However, a more qualitative approach can supplement this, by giving more information on the physiological state of the yeast cells monitored.

Yeast populations in mixed fermentations are often quantified by traditional methods such as plating using colony morphology, media composition, selective additives, and/or differential growth optima which allow distinguishing between different species [14,60,69,82,83,84]. Yeast populations can also be discriminated by using a combination of selective and non-selective culture media [26,33,52,65,71,85,86,87]. The incubation of plates at different temperatures can also be the solution to determine the population of different strains, the main example of this being *S. cerevisiae* growing at 37 °C while non-*cerevisiae* and non-*Saccharomyces* yeasts do not [10,88,89].

These methods are well understood, efficient, and rather precise. Moreover, they allow accurate interspecies discrimination using phenotypic differences, and provide information on the cell cultivability of the populations studied. However, growth time on plates involves a delay in analysis which can prove impractical when monitoring wine fermentation in real time. In addition, these culture-dependent techniques can hardly be used to monitor complex ecosystems, since some strains overcome others in culture medium [90]. They also can overlook microorganisms that grow slowly on artificial media or are present in very small amounts [72]. Moreover, sometimes, no colonies can be observed on plates, since some yeasts are in a viable but non-culturable state (VBNC) as a result of stress induced either by interaction with other yeasts or culture conditions. To confirm this state and evaluate the capacity of yeasts to recover, they are transferred into fresh liquid nutritive medium and incubated for 24-48 h, once or twice: VBNC yeasts after these cultures in ideal conditions can be cultivated again [65,91].

The need for new or adapted analysis methods that shorten the delay in obtaining information has thus emerged. Zupan et al. (2013) [92] presented a quick method to monitor the number of viable yeasts during fermentations, using microscopy and image analysis software; yeasts are observed on a hemocytometer with three settings of the same microscope to count viable, non-viable and total cells.

Flow cytometry is also an interesting technique used to enumerate microbial populations by automating the counting process, and Longin et al. (2017) [93] showed its potentiality for monitoring yeast populations during wine fermentation. As with plating, discriminating between both species studied is essential to monitor populations and highlight yeast interactions. To differentiate various strains, modified strains expressing fluorescent proteins are often used, as in the recent study by Petitgonnet et al. (2019) [46], which makes use of a GFP-modified *S. cerevisiae* to show its capacity to inhibit *L. thermotolerans* by cell–cell contact linked mechanisms. In these cases, it is necessary to verify that these modified strains have the same behavior as wild strains. This allows managing the proportion of *S. cerevisiae* in a mixed culture [94,95]. Another strategy is to use fluorescence in situ hybridization (FISH), like Wang et al. in 2014 [96], who developed specific probes and optimized conditions to monitor *S. cerevisiae* and two non-*Saccharomyces* yeasts in mixed cultures. This method is simple, rapid, and sensitive, but it involves membrane permeabilization and does not give information in real time or on cell viability. Flow cytometry can also be used to obtain extensive information on cell physiological state, as discussed below.

Authors have also used quantitative PCR to monitor yeast populations: from the isolated total DNA, amplifying a gene with species-specific primers gives the proportion of each species, and then extrapolates the population of each species in the total population. This method is rapid and very sensitive [90]. Andorra et al. (2010) [72], Wang et al. (2015) [70] studied the total population present in natural must by different techniques and showed that qPCR can be used to analyze the dynamics during wine fermentation; its advantage over culture-dependent techniques is that it takes into account non culturable yeasts. This qPCR technique was used recently to study the impact of competition between *S. cerevisiae* and other *Saccharomyces* yeasts on growth fitness and to understand the impact of nitrogen on competition between different strains of *S. cerevisiae* [58,76]. Garcia et al. (2017) [97] applied this method to monitor five non-*Saccharomyces* strains in a mixed culture, with satisfying efficiency (good specificity, sensitivity down to 10^3^ cells/mL, linearity). Another method, reverse transcription (RT)-qPCR can be used but this methodology underestimates the culturable population in wine due to the decrease of rRNA level in cells facing environmental stress (ethanol, nutrient depletion) [70]. These RNA/DNA-based methods have several advantages; they save time, they are interesting in the case of microorganisms difficult to cultivate (need for specific medium, VBNC) [70,90] and they provide precise discrimination between strains. However, these methods do not provide any information on cell physiological state or viability, since DNA from dead cells is also detected.

To study the physiological state of yeasts from mixed cultures, authors have used specific staining with different compounds and probes, allowing them to either assess viability, and even to study specific consequences on some aspects of cell physiological state. Coupled with flow cytometry (or epifluorescence microscopy), staining can provide rapid information, but choosing suitable dyes is complex and depends on both the microorganism and medium. Longin et al. (2017) [93] discussed these choices extensively in their review on flow cytometry applied to wine. For example, staining with propidium iodide (PI) can be used to evaluate the viable populations in mixed culture [46], or to study the impact of interaction mechanisms such as anti-microbial peptides on membrane permeability [91,98]. Another marker, fluorescein di-acetate (FDA) or carboxy-FDA (CFDA), can be used as an indicator of cellular vitality since it reflects enzymatic activity (esterase) [93]. This fluorophore is used by Gobert et al. (2017) [55] in cocultures involving *S. bacillaris* and *S. cerevisiae*, to monitor yeast viability during fermentations. Double stainings are often used to help discriminate live cells and assess viability during mixed fermentations [65,93,99].

Fluorophores can also be used to measure intracellular pH (pHi) since this important parameter influences metabolism and can lead to cell membrane disruption if it is modified [93]. Probes such as 5,6-carboxy-2′,7′-dichlorofluorescein diacetate (CDCF) or 5,6-carboxy fluorescein diacetate succimidyl ester (cFDA-SE) (for pH values between 3-4.5 and 4-7 respectively) combined with flow cytometry, epifluorescence microscopy or fluorescence ratio imaging microscopy (FRIM), give information on pHi [91,98], highlighting how exposure to anti-microbial peptides (AMPs) can induce a drop in pHi in non-*Saccharomyces* cells during fermentation.

Based on DNA-techniques, other methods have been developed to show a difference between live and dead cells, by using dyes able to enter cells with compromised membranes and bind to DNA, making them non amplifiable by PCR. Ethidium monoazide bromide (EMA) qPCR can then be used to monitor viable yeasts during must fermentation [70,100]. FISH can also be coupled with live/dead staining such as IP/DAPI [99], to assess the identity and viability of strains in mixed cultures at the same time.

Yeast interactions impact population dynamics and their metabolism with consequences for fermentation kinetics (more or less rapid and complete consumption of substrates (sugars, yeast assimilable nitrogen, oxygen), the production of ethanol) and for the production of other metabolites (differences in quality and content (glycerol, organic acids, aroma compounds)). Authors have usually used the same methods to monitor all these compounds: enzymatic techniques [58,65,84], or high-performance liquid chromatography HPLC [9,54,69,81,101]. Fourier transformed infrared spectroscopy FTIR can also be used since it is very convenient, simple, and rapid, but the results can lack precision. More recently, a new approach is to study more globally the metabolites produced by yeasts [46,84,102] to obtain information on the global metabolism of strains and better evaluate the role of each strain in imprinting its own metabolomic signature on the mixed culture medium.

### 2.3. Yeast Interactions: Understanding Mechanisms

Although most authors have studied population dynamics and metabolite production, some of them have focused on various mechanisms involved in yeast interactions. Interactions can be linked to modifications of medium during fermentation (decrease of nutrients or the production of inhibitory or toxic compounds) or to the direct action of a yeast on another one (with physical cell-contact, through molecules present on the cell surface). To understand these phenomena, authors can employ different strategies often used in parallel studying the impact of specific culture conditions on yeast populations and metabolisms (modification of medium composition, increasing cell contacts, suppressing cell contacts); highlighting the presence of cell contacts, modification of physiological state induced by interactions.

These different methods are described below. In addition, they are nowadays supplemented by more recent techniques: metabolomic, transcriptomic, and genomic techniques, on which we will focus in part 2 of this review and further.

Nissen et al. (2003) [26] were among the first authors to develop a strategy to understand which mechanisms are involved in yeast interactions in wine. They used modifications of culture conditions (addition of live or dead cells, addition of medium from other cultures, addition of nutrients) to highlight or not different interaction hypotheses.

In mixed culture, when certain yeast populations decline or death is observed, the first possibility is that nutrient competition occurs between both strains. To check this eventuality, authors have studied the addition of fresh culture medium or the replacement of the depleted one [26,68,84] and their impact on population dynamics.

The second hypothesis is the production of specific compounds by a strain impacting the growth of another (whether by toxic properties or other mechanisms like quorum sensing). To include or rule out this possibility, yeasts can be cultivated in pure culture in supernatants obtained from mixed culture (potentially containing toxic or inhibiting compounds (ethanol, fatty acids, peptides)), with supplementation in nitrogen sources to avoid nutrient limitation [52], or without supplementation [25,26,68]. This modus operandi can also be used to test the antimicrobial activity of peptides produced by yeasts in pure or mixed culture: more or less purified supernatants are incubated with different microorganisms [5,91,103]. This strategy makes use of a cell/medium separation technique by centrifugation, that can induce stress in yeasts (as shown by Chlup et al. 2008 [104]) and perhaps impact their metabolism.

To highlight the involvement of physical contact between two different yeast populations, biomass behaviors can be compared between a pure culture and a culture with the addition of another strain in different states and densities. Researchers have observed that the growth of non-*Saccharomyces* yeasts is not impacted by the addition of cellular debris or dead cells of *S. cerevisiae* but is immediately stopped when viable cells are added [25,26]. Thus, they showed that the presence of viable *S. cerevisiae* is necessary to influence the growth of the second yeast. In addition, they observed that a sufficiently high cellular density is required to obtain this impact, indicating the possibility of competition for space or the implication of a cell-contact mechanism. By changing the cell concentration in pure culture fermentation or the ratio of non-*Saccharomyces/S. cerevisiae* in mixed cultures, Nissen and Arneborg (2003) [25] showed that early death is not solely a consequence of high cell density or a low ratio but also of different abilities to compete for space.

Therefore, to prove that physical contact is necessary to observe a modification of population dynamics, one strategy is to suppress these contacts artificially by separating both yeast populations by a semipermeable membrane that prevents contacts between the yeasts but allows the exchange of substrates and metabolites between the two compartments. If cell–cell contacts are involved in yeasts interactions, yeast behaviors will be different in these conditions from those in mixed fermentations. Some authors used simple systems (tube or flask with dialysis membrane) with different conditions: without agitation and with a cut-off of 12–14 kDa [9,25,26,46], with agitation and a cut-off of 3.5–5 kDa [28] or 1000 kDa [28,71]. To ensure homogeneity on both sides, some authors measured the concentrations of only a few substrates and metabolites (ethanol for Nissen et al. 2003 [26], glucose and ethanol for Kemsawasd et al. 2015 [28]). Nissen and Arneborg (2003) [25] specified that fouling of the membrane was observed after 4 days of fermentation, with a difference in composition (glucose, ethanol) in both compartments. Kemsawasd et al. (2015) [28] noticed that peptides and proteins were freely transported through a 1000 kDa membrane but that 3.5–5 kDa was slightly permeable to molecules larger than 5 kDa, so that AMPs could be present in both compartments (AMPs derived from GADPH are about 8 kDa according to Branco et al. 2014 [101], AMPs studied by Albergaria et al. 2013 [103] are 2–10 kDa). More recently, Petitgonnet et al. (2019) have evaluated metabolism changes more globally by using metabolomic techniques and highlighted a notable difference in exo-metabolomes between fermentations with and without physical contact [46]. This aspect will be further developed at a later stage.

To study competition between different strains of *S. cerevisiae*, Perrone et al. (2013) [81] chose to use a partitioned reactor that had already been used in studies of bacteria cocultures (Di Cagno et al. 2009) [105]; a double culture vessel apparatus with compartments separated by a 0.45 µm membrane, and which can be stirred. The membrane allows the transfer of medium compounds and composition homogeneity is verified by HPLC for sugars, alcohol, glycerol, acids; only one difference was observed for sugars but with a value close to instrumental reproducibility. Lopez et al. (2014) [106] and Taillandier et al. (2014) [51] used a reactor composed of two jars interconnected with a hollow fiber membrane (0.1 µm). They regulated the flow between both compartments using alternating pressurization, but they observed fouling after 21 h of culture. The system proposed by Renault et al. (2013) [107] seemed to be more efficient. They designed a new double-compartment reactor; separation was ensured by a 1 µm membrane, a pump circulated the medium between both compartments through a 0.45 µm filter to homogenize it without the transfer of yeasts; it was also equipped with automatic reversal of the pumping direction to avoid clogging. All these systems used different means to separate yeasts while ensuring metabolic homogeneity, but they did not seem to allow the immediate transfer of all the metabolites in both compartments. That is why other authors took a completely different approach to investigate the involvement of cell contact in yeast interactions. Rossouw et al. (2018) [108] used a genetic system (based on *FLO* gene family) to modify cell adhesion properties and show that interspecies contacts impact population dynamics as the mechanism was called cell–cell contact by other authors. They made use of a simple sedimentation rate measurement to assess interspecific coaggregation. This macroscopic approach to aggregation dynamics could then be supplemented by microscopic analysis.

Various microscopy techniques can also be used to study different types of contact between yeasts; cell–cell contact, aggregation, or coaggregation with other cells or solids. Fluorescence microscopy with cell staining was used by Rossouw et al. (2015) [109] to highlight the co-flocculation of *S. cerevisiae* and *Hanseniaspora opuntiae* (*H. opuntiae)* in mixed cultures and to study the involvement of different *FLO* genes on flocculation. Cell staining can also be used with flow cytometry, as shown in Pérez-Torrado et al. (2017) [94], which makes use of sonication to highlight cell aggregation. Caldeira et al. (2019) [110] observed the surface of yeast in mixed cultures by atomic force microscopy and showed the existence of direct cell–cell contact. Scanning electron microscopy (SEM) can be used to highlight the aggregation of yeasts in wine with the same or other yeast species, or with solids (as shown by Govender et al. 2011 [111]).

Epifluorescence microscopy observations can also be used to study how anti-microbial peptides (AMPs) act on yeast. Branco et al. (2017) [86] used chemically synthesized AMPs, with fluorescent labelling, and added them to the medium culture of non-*Saccharomyces* yeasts. They noticed that these AMPs can enter cells and at the same time, cells that internalized these AMPs showed compromised cell membranes (PI-stained).

Other less conventional methods can also be used to study the interaction mechanisms. For example, Branco et al. (2017) [71] used immunologic testing to highlight the involvement of cell contact in AMP activity. After extracting and fractionating surface proteins from *S. cerevisiae*, they analyzed fractions by enzyme-linked immunosorbent assays (ELISA) using a specific antibody against GAPDH-derived AMPs. They showed that these AMPs accumulated on cell surfaces, suggesting a potential link between cell–cell contact mechanisms and these AMPs.

In addition to all the analysis techniques discussed previously, novel omics approaches are nowadays widely used to solve different problems and are used to describe an organism’s response to genetic or environmental changes. The impact of cell interactions on yeast metabolism is no exception, and proteomics and transcriptomics in case of yeast–yeast interactions in wine have been used extensively in recent years. Most of the studies performed to characterize the consequences of co-cultures and interactions between microorganisms on gene expression and protein synthesis have focused on comparisons with *S. cerevisiae*, an organism that has undergone extensive investigation [88,112,113]. The modulation of gene expression has been clearly observed during alcoholic fermentation [9,112,113]. Most of the genes whose expression is modified during co-cultures and interactions are involved in stress response, endocytosis, membrane biogenesis, nutrient uptake, and apoptosis [84,88,89]. Complete metabolic pathways are affected by altered gene expression, as shown by Sadoudi et al. [87], with a change in acetic acid and glycerol metabolism in *S. cerevisiae* in the presence of *Metschnikowia pulcherrima (M. pulcherrima)*. More specifically, in the case of direct cell contact between two populations of distinct species, a change in the expression of *FLO* genes has been described, leading to a modification of population dynamics [109]. We will not develop this aspect in our discussion, as the subject has recently been discussed and detailed by Conacher et al. [24].

## 3. Environmental Changes Related to Interactions and Sensory Impacts

Proteomics and transcriptomics provide insights into the impact of interactions on wine composition [24,89,112,114] but none of them has so far provided significant progress on the microbial interaction mechanisms involved. Metabolomics is a tool of choice for observing the impact of yeast interactions on the composition of the wine matrix and more interestingly, it can help unravel the as yet unknown mechanisms involved in these interactions. Analytical techniques developed for metabolomics studies allow screening hundreds of metabolites from various metabolic pathways with high-throughput techniques [115] that link the impact of yeast interactions to wine composition [102].

The literature includes various studies in which the specific composition of wine enables distinguishing between wines on the basis of fermentations with different yeast species and strain [116,117,118,119] and with single and co-cultures [46,102,120,121].

### 3.1. Metabolic Profiling

Non-targeted metabolomics studies provide a global vision of the modifications of the matrix. Through this approach, all the products from metabolic pathways affected by interactions with a second microbial population can be studied. Only a few studies aimed at understanding yeast–yeast interactions in wines have been carried out using this non-targeted metabolomic approach. Some studies have used FT-ICR-MS to explore metabolomes in wine [46,102,122]. In 2016, Liu et al. [122] studied fifteen strains of *S. cerevisiae* known to positively or negatively impact malolactic fermentation (MLF) through interaction with lactic acid bacteria. They identified a wide variety of markers such as oligopeptide and sulfur-containing peptide metabolites for each of the yeast phenotypes studied. Later, Petitgonnet et al. [46] highlighted changes in the exo-metabolome of wines from co-culture fermentation, depending on the presence or not of a physical barrier. The originality of this paper resided in the study of the physical separation of the two populations. Indeed, greater diversity of compounds was demonstrated in *L. thermotolerans* alone and contactless *S. cerevisiae*/*L. thermotolerans* modalities. Biomarkers specific to these modalities were mainly identified as involved in amino acid metabolism and carbon fixation. The general conclusion of the study shows that cell to cell yeast interaction does induce a significant change of diversity and variability in the intensity of metabolic compounds in final wine composition [46]. More recently, Roullier-Gall et al. [102] worked on the non-volatile metabolic fingerprint comparison of three different non-*Saccharomyces* species in single and co-cultures with *S. cerevisiae*. It was pointed out that the metabolite composition of wine from the co-culture did not match the assembly of two wines resulting from single yeast fermentation, an observation already made in previous studies [120,123] involving non-neutral interaction phenomena.

The majority of non-targeted works have focused on the metabolome at the end of alcoholic fermentation and therefore are not able to reveal at what stage of growth and fermentation metabolic changes occur. Fortunately, several papers have focused on the different stages of fermentation, including works from Richter’s team in 2015, who conducted alcoholic fermentation on Chardonnay must with *S. cerevisiae* [124]. Significant metabolic changes were identified at each stage of the fermentation studied. This contribution made it possible to attribute a certain regulation of yeast metabolism during fermentation to the efficiency of the glycolytic pathway, probably due to a reduced activity of several enzymes or to glucose transport. In 2018, Peng et al. [121] demonstrated the impact of bringing together two yeast populations of *S. cerevisiae* and *L. thermotolerans* at two key points of alcoholic fermentation: at the onset of early death of non-*Saccharomyces* yeast and at the end of this phase. Owing to NMR, a single culture of *L. thermotolerans* and a co-culture were discriminated based on metabolite composition variations. On the contrary, no changes could be identified when comparing the metabolome of the single culture of *S. cerevisiae* and co-culture. In addition, they highlighted that part of the metabolite composition disappeared at the end of fermentation, suggesting that metabolic changes of co-culture occur after the death of the non-*Saccharomyces* yeasts [121]. It also appears that at different sampling times, the diversity and concentration of metabolites is very different compared to previous works on single culture [124,125]. These works highlighted that sampling time is an essential point for understanding interaction phenomena. Furthermore, studies [124,126] began to explore the differences between the endometabolome and the exometabolome associated with microorganisms involved in fermentation processes to explain the mechanisms involved in interactions. However, it remains difficult to study the endometabolome because of the complexity of sampling [127]. Therefore, recent works have focused on modelling the composition of the endometabolome based on exometabolome measurements [126].

It should also be noted that the identification of compounds detected during the metabolic profiling of the non-volatile fraction of wine remains difficult at present. The complexity of wine has been widely described and still presents many shadowy areas. The databases giving the molecular compounds present in wine remain poorly supplied and do not allow identifying all the biomarkers [102,128,129].

Targeted analysis, often associated with hypothesis verification, involved detecting and quantifying known metabolites in the wine [130,131,132]. Targeted metabolomics is particularly used for studying the impact of microorganisms [118,133] and interactions [134,135] during winemaking. In the context of leavening different starters to carry out fermentations in the best possible way and with the objective of managing the final quality of Syrah wines, Minnaar et al. [134] were interested in understanding the microbial interactions involved. It would appear that microbial interactions affect the production of polyphenolic compounds including anthocyanins, flavonols, and phenolic acids. Different combinations of starters were studied, involving respectively a strain of *S. cerevisiae* with *M. pulcherrima* or *Hanseniaspora uvarum (H. uvarum)*, *S. cerevisiae* and one of the non-*Saccharomyces* with a LAB strain, and *S. cerevisiae* with a lactic acid bacteria (LAB) with a single starter of *S. cerevisiae*. They identified for the mixed starter of *M. pulcherrima/S. cerevisiae* a decrease in the amount of gallic acid and of caffeic acid for *H. uvarum/S. cerevisiae*. Similarly, Nardi et al. [135] studied the impact of a co-culture of *S. cerevisiae* with a strain of *T. delbrueckii* in combination or not with a strain of *O. oeni*. They were able to demonstrate that among the extracted compounds, involved in the discrimination of the different conditions, there was an increase in certain metabolites such as amino acids like alanine and threonine, and at the same time a decrease in deleterious compounds such as acetic acid. These works described the consequences of the presence of several yeast populations in co-cultures but did not describe the mechanisms involved in the underlying interactions.

### 3.2. Volatilome

The yeast metabolome does not consist entirely of compounds from the non-volatile fraction. In fact, a wide variety of volatile organic compounds (VOCs) are released during the winemaking process and enrich the total wine composition. These compounds produced by microorganisms are grouped together under the term “volatilome” [136]. The composition of the volatilome is related to the species [47,137] and the strains of microorganisms [79,138] used to conduct alcoholic fermentation. In addition, the interactions occurring in consortia or co-cultures also influence the VOC composition of wines [139]. Many VOCs participate in the aromatic profile of wines, as developed later in the discussion [140,141,142]. Unfortunately, as with non-volatile omics studies, most volatile studies focus on the impact of yeast interaction on the final VOC composition of co-cultured wines, but few explain the mechanisms of interactions and allow us only to hypothesize about the nature of yeast interactions.

VOCs can be produced by yeasts metabolizing sugars and amino acids and belong to different families of compounds including esters, higher alcohols, medium fatty acids, or aldehydes. Among these fermentation aromas, esters are widely represented, comprising acetate esters and ethyl esters. In the case of co-cultures between *S. cerevisiae* and non-*Saccharomyces* yeasts, the concentration of the major esters is mainly increased. For mixed crops in which *M. pulcherrima*. [47,143] and *H. uvarum* [144] are involved with *S. cerevisiae*, the concentration of esters increases, for example that of phenylethyl acetate. For the most part, mixed crops, including *S. bacillaris,* present higher concentrations of total esters [79,138], including ethyl octanoate or isoamyl acetate [14] compared to pure *S. cerevisiae* fermentation conditions. Conversely, when focusing on Muscat wort, Gobert et al. [55] showed a decrease in the production of isoamyl acetate in sequential fermentations of *S. bacillaris* and *S. cerevisiae*. Similarly, some esters such as isoamyl acetate and ethyl octanoate are detected in higher concentration in wines from co-fermented wort with *S. cerevisiae* and *Torulaspora delbrueckii* (*T. delbrueckii)* [145] or *L. thermotolerans* [45] and in lower concentrations in other studies [39,146] (Table 2). This different impact of co-culture on esters concentration can be explained by the use of various yeast strains and matrix [147]. In the same way, co-culture may increase the content of higher alcohols found in wines. This has been thoroughly described by Sadoudi et al. [47] for the mixed fermentations conducted with *Candida zemplinina* (*C. zemplinina)* and *S. cerevisiae*, Escribano-Viana et al. [37] for *T. delbrueckii* and *S. cerevisiae* and Englezos et al. [79,138] for *S. bacillaris* and *S. cerevisiae*. However, others studies focusing on mixed-cultures with *S. bacillaris* and *S. cerevisiae* have shown a lower concentration of certain alcohols such as 2-phenylethanol and methyl butanol [55,148]. Fatty acids have been found in lower concentrations in most of the co-cultures studied using non-*Saccharomyces* [79,135] except for the couple with *S. bacillaris* [14] and *C. zemplinina* [36] (Table 2). Varietal aromas can also be released by yeasts through the action of cleavage enzymes on odorless precursors present in the must such as terpenes, sulfur compounds, and volatile phenols [139,149]. The combination of yeast populations with a diversity of metabolism as enzymatic activity during fermentation can impact on their diversity and concentration [150]. Finally, one of the impacts studied most on VOC families in co-cultures are terpenes. The terpenes found in wines are mostly linalool, geraniol, and citronellol. In most cases they are found in higher concentrations in wines from mixed culture alcoholic fermentation [36,147].

Although most studies describe the impact of interactions on VOC composition in fermentation in co-culture, some papers have tried to explain the interaction mechanisms associated with these compositional changes. Sadoudi et al. [47] highlighted interaction mechanisms for three couples of yeast by comparing VOC concentration in simple cultures and mixed cultures. The concentration of some terpenols such as 𝜷-damascenone doubled in the co-culture of *M. pulcherrima/S. cerevisiae* showed a positive interaction between both strains. The synergetic effects of *T. delbrueckii/S. cerevisiae* have also been revealed in co-culture, exhibiting an increase in the content of terpenols, C6 compounds and 2-phenylethanol, suggesting a cumulative effect of both yeast metabolisms related to biomass. Negative interactions for *C. zemplinina/S. cerevisiae* were highlighted by showing a decrease in the production of farnesol in co-culture, in comparison to a pure culture of *C. zemplinina*, which could be used as a modulator of gene expression. As mentioned above, later, in 2017, the same team showed that the presence of *M. pulcherrima* induced a change in the gene expressions involved in the metabolism of acetic acid of *S. cerevisiae* in co-cultures [87]. Later, Petitgonnet et al. [46] showed the importance of cell–cell contact in VOC composition in the case of *S. cerevisiae* and *L. thermotolerans* co-culture. The study compared VOCs from wines in pure culture, conventional contact co-culture, and co-culture physically separated by dialysis rod. It was pointed out that ester and fatty acid concentrations were higher in co-culture without cell contact. Similarly, other studies [12,55] have focused on nutrient sources as well as on competition for nutrients between species in the case of co-fermentation together with their impact on VOC composition. In sequential fermentations nutrient sources, such as nitrogen, are reduced at the time of *S. cerevisiae* inoculation. Gobert et al. [55] highlighted that, in the case of sequential fermentation, amino acids such as leucine are consumed by *S. bacillaris* before *S. cerevisiae* inoculation. However, leucine is the precursor of VOCs including isoamyl acetate and 2-methylbutanol mainly synthesized by *S. cerevisiae*. The depletion of leucine before *S. cerevisiae* inoculation would therefore lead to under-expression of these VOCs in wines. On the other hand, this paper showed a possible synergetic effect between *S. bacillaris* and *S. cerevisiae* for the synthesis of isobutyl alcohol [55].

Although most of the work has focused on the interaction between *S. cerevisiae* and non-*Saccharomyces* yeasts, few papers have explored other types of mixed fermentations. King and Capece studied the impact of co-cultures of different strains of *S. cerevisiae* on the volatilome [123,158]. In 2008, King found a positive variation in the thiol 3-mercaptohexan-1-ol (3MH) composition of Sauvignon Blanc wines in some of the co-cultures of *S. cerevisiae* strains studied [123]. Later, Escribano et al. [137] studied the co-culture of three yeasts in sequential fermentations including two non-*Saccharomyces*, *T. delbrueckii* and *L. thermotolerans* yeasts with *S. cerevisiae* yeast. Similarly, consortia including several non-*Saccharomyces* species have been considered [64,157]. Interestingly Padilla et al. [157] showed that a consortia of four non-*Saccharomyces* strains in mixed culture with three *S. cerevisiae* strains led to a decrease in total acids and did not affect the synthesis of other compounds in comparison to a pure fermentation with commercial *S. cerevisiae*.

Thus the yeast species [47] and the yeast strain [79,138] are the determining factors for the final wine VOC composition [159]. Nevertheless, the production of most of these VOCs also depends on different biotic and abiotic factors. It has been widely described that the composition of the matrix, and more particularly nitrogen sources [59], and the presence of VOC precursors have an impact on the production of VOCs [152]. In addition, the great variability in yeast inoculation protocols such as simultaneous and sequential fermentation [147], the time of adding *S. cerevisiae* in the case of sequential inoculation [39], population ratios [45,153], and environmental conditions [160] appears to play an essential role in yeast growing characteristics and subsequent VOC composition. Furthermore, the formation of these compounds occurs throughout the fermentation process. That is why some studies have aimed at identifying differences in VOC content based on the fermentation time [145]. They showed that the concentration of total esters increased by 40% from the beginning to the end of alcoholic fermentation. Another example was described by Escribano et al. [137] with the variation of the concentration of ethyl lactate for the ester formation due to an increase of higher alcohols in the medium. All of these factors induce additional variability between the different studies and observations [122,144].

Taken as a whole, the results of the various studies of volatile compounds show divergences. The approaches to data analysis are highly diverse, thus adding to the factors described previously that influence VOC composition. Most studies have attempted to describe differences between co-cultures and single fermentations of *S. cerevisiae* but some studies have compared the impact of these co-cultures with non-*Saccharomyces* single cultures, which may lead to differences in interpretation. Studies of VOCs in co-cultures have mainly focused on providing a descriptive approach and only a few of them have tried to explain the mechanisms leading to differences in VOC composition.

### 3.3. Sensory Impact

For many years, the impact of micro-organisms on the sensory component of wine has been addressed by many researchers and professionals in the wine sector. These studies have aimed at characterizing the individual impact of a *S. cerevisiae* or a non-*Saccharomyces* yeast strain on the aromatic [149,161,162,163], visual [164] and taste [165] profiles of wines. This approach is also increasingly applied to pairs or consortia of microorganisms involved in alcoholic fermentation [64,157]. In most cases the discrimination of wines fermented by more than one species of microorganism from those fermented by a single species is described. For example, Gobbi et al. [33] attributed more spicy and acidic notes to the co-culture in commercial white grape must compared to the single culture of *S. cerevisiae*. Barbera wine was studied after alcoholic fermentation assessed by *T. delbrueckii* and *S. cerevisiae* and showed a lower intensity in attributes such as floral and red fruit aromas, as well as a change in color, becoming more intense compared to *S. cerevisiae* in pure culture [135]. The same year, Varela et al. (2017) [156] showed that wines inoculated with *M. pulcherrima* and *S. cerevisiae* were closer to uninoculated wines and associated with high scores for positive attributes such as red fruit aroma and overall fruit aroma, which are found in a minority in wines from *S. cerevisiae* in pure culture.

More specifically, in various studies it appears that the sequential fermentation path has a greater impact on the sensory profiles of wines than simultaneous inoculation [33,145,148]). Benito et al. [40] showed in a comparison between sequential fermentation and simultaneous inoculation, *S. cerevisiae* and *L. thermotolerans*, that general acidity and overall impression were increased and described as characteristic of sequential fermentations. In 2015, the same research team also attempted to determine the impact of mixed starter wines with a non-*Saccharomyces* strain on the quality of Riesling wines [38]. As observed previously, the general impression was described as better in the case of sequential fermentations with respect to a single culture control of *S. cerevisiae*. The *M. pulcherrima/S. cerevisiae* pair was discriminated by the terms citrus/grapefruit and pear while *L. thermotolerans/S. cerevisiae* pair was associated with peach/apricot [38]. This last example shows that different aromatic notes are detected from the same matrix couples involving the same strain of *S. cerevisiae,* but with different non-*Saccharomyces* strains. Binati et al. [14] were also able to discriminate wines of Pinot Grigio from different sequential fermentations involving *M. pulcherrima* and *S. bacillaris* by different VOCs such as higher alcohols and esters. King et al. [123] highlighted a difference in sensory profiles between two co-inoculations of *S. cerevisiae* involving different strains characterized by box hedge and floral aromas and with different blends of two simple cultures of the same strain themselves described by the terms white vinegar and bruised apple. There is therefore an impact of co-cultivation and therefore interactions between populations on the sensory profile in addition to the strain effect. On the contrary, when studying two strains of *T. delbrueckii* in mixed culture with *S. cerevisiae,* Azzolini et al. [146] found no differences in the sensory profile with either pair. However, complexity and persistence were found to be increased in mixed cultures compared to the single culture of *S. cerevisiae*. Likewise, Liu et al. [122] mentioned that the impact of cultivar type was greater than the strain effect. Indeed, as pointed out earlier, the matrix plays an important role in the sensory profile. Hu et al. [144] confirmed the greater role of the matrix specifically the grape varieties with the observed decrease in the vegetal component in a Cabernet Sauvignon wine as opposed to an Ecolly wine fermented with the same mixed starter.

These latest works were carried out by studying VOC composition and the sensory aspect of wines in parallel; however, few of them focused on linking these two aspects. Hu et al. [144] established that the compounds that mainly contributed to tropical fruit and floral aspects of sequential fermentation that involved *H. uvarum/S. cerevisiae*, were C13 norisoprenoids, terpenes, and acetate esters, while the temperate fruit notes were generated mostly by ethyl esters. Similarly, Nisiotou et al. [49] identified an association between a higher concentration of ethyl ester and the fruity aroma of wine, as previously discussed by Lytra et al. [142] for simultaneous and sequential cultivation using *L. thermotolerans.* They likewise noted a correlation between acetate esters and the floral aroma descriptor. In the work by Renault et al. [145] on the sequential fermentation of *T. delbrueckii* and *S. cerevisiae,* the over-expression of four esters (ethyl propanoate, ethyl isobutanoate, ethyl dihydrocinnamate, and isobutyl acetate), described as minor, was highlighted. Moreover, the wine was characterized and differentiated from other modalities by fruity aromas and greater complexity. Then, they added at equal concentration the sequential condition and the single culture of *S. cerevisiae* to validate the impact of these esters on the wine sensory profile. This made it possible to highlight the sensory impact of these esters and attribute them the role of aromatic biomarker. However, it should be noted that only one of its esters was present at a concentration above its detection limit in wine. Therefore, it is suggested that the other esters also lead to aromatic modulation through different interaction phenomena. Among the four esters, ethyl propanoate and isobutyl acetate were described by several studies as enhancers of fruity notes [140,142,166].

The sensory aspect of the various studies mentioned above shows changes in the sensory profile, but they do not address or make it possible to understand or explain the mechanisms of interaction between the populations involved in alcoholic fermentation. Most authors have focused more on showing a change in the sensory profile of the wines in the case of co-culture. Indeed, sensory contribution remains complicated to integrate into a process of understanding the mechanisms of interaction, since no VOC or family of VOCs can explain the aromatic profile of a wine. It is still unclear what contribution they make to the aromatic notes. Despite trends and correlations, there is not always a mirror effect between chemical composition and sensory profiles due to the existence, among other things, of interactions between these volatile aromatic compounds and non-odorous volatile compounds to form aromas [141,167,168]. These interactions between VOCs were confirmed in a very recent publication by Mc Kay et al. [169]. Associated with these interactions, various factors can induce a mismatch between the volatile composition and the sensory profile of wines, such as the detection threshold [159,163,169,170], or the masking of certain flavors associated with volatile compounds by others, as suggested by Benito et al. [38] cited above. Higher alcohols have already been described as being able to mask these fruity notes [171]. Finally, in view of the diversity of volatile aromatic compounds, many of them participate in the same aromatic note [172]. Sensory evaluation also provides different information depending on the approach selected (description, comparison, preference, or determination of product quality), [160] and the panel of selected juries (expert or naive) [173]. Sensory analysis therefore remains an essential tool to qualify the impact of co-cultures on the final product, but it does not provide information on the interaction mechanisms that may occur.

## 4. Conclusion and Perspectives

This review presented the state-of-the-art of yeast–yeast interactions in wine and highlighted the difficulties of studying the mechanisms involved in these phenomena. The impact of co-culture on the final matrix is now well-known but little is understood about how this happens. Indeed, it appears that all the works presented distinct methodologies, mainly in terms of biological material with the use of different yeast species and strains, leading to a plethora of results specific to each pair. Therefore, understanding these mechanisms requires further studies at this level by combining the observation of a target mechanism with the use of different strains belonging to the same species in order to draw solid conclusions. Different matrices and the application of abiotic factors also remain a major source of diversity in the results. All these variations make it difficult to generate complementary and comparable results capable of leading to conclusions that unravel the mechanisms involved in these interactions.

During our research it became apparent that quorum sensing in yeast remains unexplained and unproven, making this gap in knowledge an important path of investigation, as discussed by Winters et al. 2019 [174]. Quorum sensing interactions have been proposed many times as a hypothesis by the authors [26,175], but neither have confirmed it. Although the direct impact of certain specific QS molecules on non-*Saccharomyces* growth has already been studied [59] when in high concentrations, experiments under conditions closer to those of winemaking have yet to be conducted. It also appears that the study of different types of interaction mechanisms such as cell–cell contact, for example, presents many contradictory results because of the use of different systems aimed at separating the different populations involved. The use of a double compartment bioreactor with a membrane making it possible to homogenize the surrounding medium in both compartments, while not denaturing the separation of microorganisms, seems to be a good strategy for understanding mechanisms. In addition, strategies at the molecular level can be considered to elucidate these mechanisms. For example, the creation of mutants of target genes that are presumed to be involved in mechanisms due to interactions such as cell–cell contact, and parietal genes, could be one avenue of investigation. Competition for nutrients is still difficult to assess when discriminating between the consumption of a nutrient by one or another of the populations involved. Monitoring this catabolic activity could be carried out, for example, by tagging amino acids for nitrogen competition. It was observed that, overall, the majority of the studies were essentially descriptive and failed to capture the interactions and their mechanisms. Technological deadlocks remain that must be overcome. The metabolomic approach is a real tool of choice and evokes metabolic pathways associated with changes in the composition of the metabolome, however, this requires further development of databases related to yeast metabolism in wine. With respect to representing the end result of all the metabolic and regulatory interactions that lead to metabolic changes, monitoring metabolic flows, currently called “fluxomics,” is one of the avenues to be considered with, for example, isotopic labelling of metabolites. In addition, a question arises as to the representativeness of targeted approaches that allow the quantification of target compounds in relation to the totality of the metabolites produced. A non-targeted approach, as suggested by Suklje et al. [176], may further explain the metabolic changes that occur. Transcriptomics, an indispensable approach for understanding gene expression under established environmental conditions, is still limited from the non-*Saccharomyces* perspective, as the genomes are poorly sequenced. Data mining is also of great importance since it can lead to the establishment of models of microbial behavior in response to different individual or combined parameters. An integrated approach combining different omics techniques is a strategy of choice that was recently described by Lawson et al. [177] with the objective of better understanding the mechanisms that direct interactions within a consortium of microorganisms. However, it should be taken into account that these approaches provide additional information and do not always lead to a general combined conclusion.

## Figures and Tables

**Table 1:** Diversity of methodologies and results in *S. cerevisiae*/*Lachancea thermotolerans* interaction experiments.

**Species**				***S. cerevisiae/Lachancea thermotolerans***																																													
**Matrix**				**Synthetic Medium**																									**Grape Juice/Must (Unspecified Color)**																				
				**SGJ**		**WYPD**				**SGJ**			**WYPD**								**SGJ**								**Grape Juice**					**Grape Must**									**Grape Must**						
**Sugar Initial Concentration (g/L)**				210 (Glc)		200 (Glc)				210 (Glc)			200 (Glc)								200 (Glc 100 Fru 100)								162					160									231						
**YAN/PAN (mg/L)**																																											PAN 154/NH3 22						
**Temperature (°C)**				25		25				25			25								25								25					20									25						
**Oxygen**				+/-		+/-		+		+/-			+								-	+			++		+++		+/-		+			+									-						
**Inoculation delay between two strains (h)**				0		0				0			0								0								0		0			0		24		48		72			0						
**Ratio Sc/NS**				1:1		1:1				1:1			1:1								1:10								1:1		1:1			1:1									1:1			1:100			1:10,000
**Sc population**			**Max**	2		1		1		2			1			1					3	3			3		3		2		2			1		5		7		7			2			4			15
			**Dominance**	+		=				+			+ 1			+ 1					+								+		+			+ 1		+ 2		+ 7		+ 12			+ 1			+ 10			+ 15
			**Decrease**	-		2.5		2.5		-			-			-					-	3			3		3		6		-			-		-		-		-			-			-			-
**NS population**			**Max**	2		1		1		2			1			1					1	1			1		1		2		2			3		2		5		3			1			3			3
			**Decrease**	3		2.5		2.5		3-6			1			1					-	-			-		-		3		4			3		6		6		10			3			10			15
			**Detectable End**	Low 6						Low 6-10			No 6			Low 6/Yes													Low 4		Low 6			No 7		No 8		Low 8 No 30		Low 30			No 15			No 22			No 22
**Fermentation completion**				10						10			6			5													10		10			+		+		+		+			+						
**Ethanol**																					=	- -			- -		- -							- -		- -		- -		=			- -						
**Glycerol**																					++	- -			- -		- -							=		++		++		++			++						
**Organic acids**			**TA**																															++		++		++		++			++						
			**VA**																															- -		- -		- -		- -			- -						
			**LA**																															++		++		++		++									
			**AA**																																														
**Impacting Factors**																					OX/SPE								OX					DEL									RAT/SPE						
**Interaction Mechanisms**				No TOX/No COMP/CCC		No TOX/No COMP/No QS/CCC				COMP			TOX/CCC								COMP								No TOX/COMP																				
**Reference**				[25]		[26]				[27]			[28]								[29]								[30]					[31]									[32]						
**Species**				***Saccharomyces cerevisiae/Lachancea thermotolerans***																																													
**Matrix**				**Red Grape Juice/Must**																																													
				**Sangiovese - Cabernet Sauvignon 1:1**											**Sangiovese**								**Tempranillo**					**Tempranillo**					**Shiraz**						**Tempranillo**			**Tempranillo**						**Pinot Grigio**	
**Sugar Initial Concentration (g/L)**				254											222								249					226					280 (Glc 140 Fru 140)						245			226						236	
**YAN/PAN (mg/L)**				PAN 160/NH_3_ 36																			PAN 167					PAN 241					YAN 170 (supp)						YAN 181			PAN 333						YAN 235	
**Temperature (°C)**				20							30				25								20					25					25									25						22	
**Oxygen**				++											+								+					-					++						+			+						+	
**Inoculation delay between two strains (h)**				0											0				48				96					96					96						72			72						48	
**Ratio Sc/NS**				1:10											1:10				1:10				10:1					1:1					1:1						1:1			1:1						1:1	
**Sc population**	**Max**			4							1				4				7				6					8																					
	**Dominance**			-																																													
	**Decrease**			-							-				4				7				-					-					-									-						-	
**NS population**	**Max**			4							4				2				2				4					4																					
	**Decrease**			18							4				4				4				4					4					+						+			5						3	
	**Detectable End**										No 7				Low 10				Low 10				No 8					Low 10					No 7						No 6									Low 5	
**Fermentation completion**				24							13												12					10					17															14	
**Ethanol**				- -							=				- -				- -				- -					-					=						- -			- -						- -	
**Glycerol**				++							+				++				++				++					++											+			=						++	
**Organic acids**	**TA**			++							++				++				++																														
	**VA**			+							- -				++				++														++						= or ++										
	**LA**			++							+				++				++									++														++						+/-	
	**AA**																						- -					- -														- -						++	
**Impacting Factors**				TEMP/DEL/GRA/REAC																													SPE						SPE									SPE/NS	
**Interaction Mechanisms**																																																	
**Reference**				[33]																			[34]					[35]					[36]						[37]			[13]						[14]	
**Species**				***Saccharomyces cerevisiae/Lachancea thermotolerans***																																													
**Matrix**				**White Grape Juice/Must**																																													
				**Grape Must**					**Riesling**			**Emir**								**Airen**						**Sauvignon Blanc**				**Airen + Catechin Addition**		**Chardonnay**					**Riesling**				**Pedro Ximenez/Sun-Dried**						**Grape Must**		
**Sugar Initial Concentration (g/L)**				224					237			205								245						217				234		218					225				487						212		
**YAN/PAN (mg/L)**				PAN 154/NH3 22					PAN 147											PAN 177						YAN 170 (supp)											YAN 250 (supp)										251		
**Temperature (°C)**				25					20			18								25						15				25							20				22						20		
**Oxygen**				-								+								+						+				-		-			++		+				+						+		
**Inoculation delay between two strains (h)**				0	24		48		48			0		24			48	72		0				96		96				168		0					48				0						24		
**Ratio Sc/NS**				1:10	1:10		1:10		10:1			1:1								1:2.5				1:2		1:1				1:1.3		1:10					1:1				1:5			1:20	1:50		1:1		
**Sc population**		**Max**		2	1		2		4			4								4				8								4			2		4										3		
		**Dominance**		+ 11	-		-					+																													-			-	-		+		
		**Decrease**		-	-		-		-			6		6						-				-								6			6		14										-		
**NS population**		**Max**		1	2		2		2															4								0					0				6			6	6		1		
		**Decrease**		11					2			4		6						+				4		+				+ or -		4			6		4				-			-	-		5		
		**Detectable End**		Low 18					No 15			Low 7		Yes						No 6				No 8		No 14						No 6			No 8		No 6												
**Fermentation completion**				+	-		-		+			7		9						10				14		19						10			12		+				-			-	-		16		
**Ethanol**				- -	- -		- -		-			=		- -						-				- -		=				=		=			- -		=				- -			- -	- -		- -		
**Glycerol**				+	++		++		++											-				++								=			=		++				- -			- -	- -				
**Organic acids**		**TA**		++	++		++					++		++																- -							=												
		**VA**		- -	- -		- -					++		+++												- -				- -																			
		**LA**		++	++		++		+											++				+++						++							++												
		**AA**							=											=				- -		-						=			=		=				- -			- -	- -				
**Impacting Factors**				TEMP/DEL/GRA/REAC					SPE			DEL														SPE				MED		OX/SPE					DEL				RAT								
**Interaction Mechanisms**																																COMP															CCC/COMP		
**Reference**				[33]					[38]			[39]								[40]						[41]				[42]		[43]					[44]				[45]						[46]		

**YAN** = yeast assimilable nitrogen/**PAN** = primary amino nitrogen. **Sc** = *S. cerevisiae*/**NS** = Non-*Saccharomyces.* **TA** = total acidity/**VA** = volatile acidity/**LA** = lactic acid/**AA** = acetic acid. **SGJ** = Synthetic Grape Juice/**Glc** = Glucose/**Fru** = Fructose/**WYPD** = Yeast Peptone Dextrose medium modified for wine fermentation/**Supp** = with supplementation. **Oxygen**: **-** = anaerobia, +/- semi-anaerobia, low oxygenation, + = semi-anaerobia, ++ aerobia, +++ aerobia, with higher oxygenation. **Population columns**: **Max =** maximal population reached by day x/**Dominance**: + = dominance of *S. cerevisiae*, = similar populations, “x” = dominance obtained after x days/**Decrease** = decrease since day x/**Low** = low population since day x/**No** = population not detectable since day x/**Yes** = population still detectable at day x. **Fermentation completion**: x = reached at day x, +/- = reached/not reached during experimentation. **Ethanol, glycerol and organic acids** are compared to Sc pure culture: +++ very high increase, ++ high increase, + increase, +/- slight decrease, = no change, - decrease, - - high decrease. **Impacting factors**: inoculation delay (**DEL**), inoculation ratio between *S. cerevisiae* and non-*Saccharomyces* yeast (**RAT**), yeast species (**SPE**), yeast strain *S. cerevisiae* (**SC**) or non*-Saccharomyces* (**NS**), medium composition (**MED**), grape nature (**GRA**), temperature (**TEMP**), oxygenation (**OX**), type of reactor (lab, pilot, industrial) (**REAC**). **Interaction mechanisms**: involvement of quorum sensing mechanisms (**QS**), toxic compounds (including ethanol, antimicrobial peptides) (**TOX**), competition for nutrient (including oxygen) (**COMP**), cell-cell contact mechanisms (**CCC**)/**No** = mechanism involvement has been ruled out by the study.

**Table 2:** Impact on volatile organic compounds of different mixed starters.

Mixed Culture	VOCs Families	VOCs Impacted by Interactions	Impact	Inoculation Protocol	Mixed Culture Compared to	Matrix	SA	Ref.
*S. cerevisiae/S. cerevisiae*	**esters**	acetate esters	-	sim/blend	S	Sauvignon blanc	x	[123]
		ethyl dodecanoate	+					
	**thiols**	3-mercaptohexan-1-ol	+					
*C. zemplinina/S. cerevisiae*	**thiols**	3-mercaptohexan-1-ol	+	sim	S	Sauvignon		[151]
			-	seq	S and NS	Sauvignon		[47]
	**higher alcohols**		+	seq	S and NS	Sauvignon		[47]
	**terpenes, lactones, norisoprenoids**		-	seq	S and NS	Sauvignon		[47]
	**lactones**		+	seq	S	Shiraz		[36]
	**ethyl esters, MCFA**		+	seq	S	Shiraz		[36]
*L. thermotolerans/S. cerevisiae*	**higher alcohols**		+	sim	S and NS	Pedro Ximenez		[45]
				seq	S and NS	Pinot Grigio	x	[14]
		1-propanol, methionol		seq	S	Tempranillo		[37]
		2-methyl butanol, 3-methyl butanol, iso-butanol		sim/seq	S	Emir	x	[39]
			-	seq	S and NS	Muscat		[46]
	**esters**	2-phenyl ethanol acetate, ethyl acetate, isoamyl acetate, isoamyl decanoate	+	sim	S and NS	Pedro Ximenez		[45]
		ethyl octanoate		seq	S and NS	Pinot Grigio	x	[14]
		2-phenylethyl acetate, isobutyl acetate, hexyl acetate		sim/seq		moschofilero	x	[147]
				seq	S	Shiraz		[36]
				sim/seq	S	Ecolly, Cabernet Sauvignon	x	[144]
		isoamyl acetate	-	sim/seq	S	Emir	x	[39]
				seq	S	Synthetic must with precursors		[152]
				seq	S and NS	Muscat		[46]
	**fatty acids**		+	seq	S and NS	Muscat		[46]
			-	seq	S and NS	Pinot Grigio	x	[14]
		ethanoic acid		sim	S	synthetic must		[153]
	**terpenes**	geraniol, citronellol	+	sim	S and NS	Pedro Ximenez		[45]
				seq	S and NS	Pinot Grigio	x	[14]
		geraniol, damascenone		sim/seq		moschofilero	x	[147]
		geraniol, linalool, alpha terpinene		seq	S	Shiraz		[36]
		linalool, geraniol		seq	S	Synthetic must with precursors		[152]
	**aldehydes/lactones**		-	sim	S and NS	Pedro Ximenez		[45]
	**volatile phenols/norisoprenoïds**		+	sim	S and NS	Pedro Ximenez		[45]
*H. uvarum/S. cerevisiae*	**higher alcohols**		-	seq	S and NS	Rondo, Bolero, Regent	x	[122]
				seq	S	Malbec		[154]
	**esters**		+	sim/seq	S	Ecolly, Cabernet Sauvignon	x	[144]
		ethyl acetate, 2-phenylethyl acetate		seq	S and NS	Rondo, Bolero, Regent	x	[122]
	**fatty acids**		-	seq	S	Malbec		[154]
*T. delbrueckii/S. cerevisiae*	**higher alcohols**	2-phenyl ethanol, methionol	+	seq	S	Chardonnay, Soave, Vino Santo	x	[146]
		2-phenyl ethanol, 1-butanol, methionol		seq	S	Tempranillo		[37]
		2-phenyl ethanol		seq	S	Sauvignon		[47]
				seq	S and NS	Pinot Grigio	x	[14]
		phenlethyl alcohol		sim/seq	Sc	Cabernet sauvignon		[155]
	**esters**	acetate esters (2-phénylethyl acetate, isoamyl acetate, hexyl acetate, isobutyl acetate) and ethylesters (ethyl lactate, ethyl octanoate, ethyl hexanoate))	+	seq	S	Sauvignon, Syrah	x	[145]
				sim/seq	S	Cabernet sauvignon		[155]
		acetate esters		sim	S	Barbera	x	[135]
		acetate esters (isoamyl acetate) and ethylesters (ethyl octanoate)	-	seq	S	Chardonnay, Soave, Vino Santo	x	[146]
		acetate esters		seq	S	Sauvignon		[47]
	**fatty acids**	hexanoic acid and octanoic acid	-	seq	S	Chardonnay, Soave, Vino Santo	x	[146]
				seq	S and NS	Pinot Grigio	x	[14]
				sim/seq	S	Cabernet sauvignon		[155]
				sim	S	Barbera	x	[135]
	**terpenes**	geraniol, linalool, alpha terpinene	+	seq	S	Shiraz		[36]
		linalool, geraniol		seq	S	Synthetic must with precursors		[152]
		geraniol, nerolidol, farnesol		seq	S	Sauvignon		[47]
				seq	S and NS	Pinot Grigio	x	[14]
				sim/seq	S	Cabernet sauvignon		[155]
	**volatile phenols**	4-vinylphenol, 4-vinylguaiacol	-	seq	S	Chardonnay, Soave, Vino Santo	x	[146]
				sim/seq	S	Cabernet sauvignon		[155]
*S. bacillaris/S. cerevisiae*	**higher alcohols**		+	seq	S	Chardonnay, Riesling, Muscat, Sauvignon blanc		[79]
				seq	S	Barbera		[138]
				seq	S and NS	Pinot Grigio	x	[14]
		2-phenylethanol	-	sim	S and NS	Montepulciano	x	[148]
				sim/seq	S	Kotsifali/Mandilari		[49]
		isobutyl alcohol 1-hexanol increase, methyl butanol decrease	+/-	seq	S	Muscat		[55]
	**esters**		+	sim/seq	S	Kotsifali/Mandilari	x	[49]
				sim	S and NS	Montepulciano	x	[148]
				seq	S	Chardonnay, Riesling, Muscat, Sauvignon blanc		[79]
				seq	S	Barbera		[138]
		ethyl octanoate, isoamyl acetate		seq	S and NS	Pinot Grigio	x	[14]
		isobutyl acetate phenylethyl acetate, isoamyl acetate	+/-	seq	S	Muscat		[55]
	**fatty acids**		+	seq	S	Chardonnay, Riesling, Muscat, Sauvignon blanc		[79]
	**volatile phenols, carbonyl compounds**		+	seq	S and NS	Pinot Grigio	x	[14]
			-	seq	S and NS	Pinot Grigio	x	[14]
*M. pulcherrima/S. cerevisiae*	**higher alcohols**	1-propanol, methionol	+	seq	S	Tempranillo		[37]
				sim	S	Merlot	x	[156]
				seq	S and NS	Pinot Grigio	x	[14]
		phenylethyl alcohol, isobutyl alcohol		seq	S	Muscat		[55]
			-	sim	NS	synthetic must		[153]
	**esters**		+	sim	S	Merlot	x	[156]
				seq	S	Shiraz		[36]
				seq	S and NS	Pinot Grigio	x	[14]
				seq	S	Muscat		[55]
				seq	S	Sauvignon		[47]
	**fatty acids**		+	seq	S	Sauvignon		[47]
	**terpenes**		+	seq	S and NS	Pinot Grigio	x	[14]
		geraniol, linalool, alpha terpinene		seq	S	Shiraz		[36]
		linalool		seq	S	Sauvignon		[47]
	**volatile phenols, carbonyl compounds**		-	seq	S and NS	Pinot Grigio	x	[14]
	**sulfur compounds**	dimethylsulfide, ethanethiol, sulfure hydroxyde	+	sim	S	Merlot	x	[157]
Mix of four non-*Sacch* and three *S. cerevisiae*	**total acids**		-	seq	S and NS	synthetic must	x	[157]
	**others compounds**		=					
*S. cerevisiae/* non*- Sacch and T.d*		**methionol, ethyl lactate**	-	seq	S	Tempranillo		[37]
Mix of five non*-Sacch/S. cerevisiae*				seq		synthetic must Sauvignon blanc	x	[64]

**Ref.:** References ***S. cerevisiae*:** *Saccharomyces cerevisiae* ***C. zemplinina*:** *Candida zemplinina* ***L. thermotolerans*:** *Lachancea thermotolerans* ***H. uvarum*:** *Hanseniaspora uvarum* ***T. delbrueckii*:** *Torulaspora delbrueckii* ***S. bacillaris*:** *Starmerella bacillaris* ***M. pulcherrima*:** *Metschnikowia pulcherrrima*. **Impact on VOCs concentration: -:** decrease **+:** increase **+/-**: two cases are encountered **=:** no change **sim**: simultaneous/**seq**: sequential **S**: *S. cerevisiae*/**NS**: non-*Saccharomyces*. **SA**: sensory analysis. **VOCs**: volatile organic compounds.

## Supplementary Materials

The following are available online at https://www.mdpi.com/2076-2607/8/4/600/s1, Table 1S: Diversity of methodologies and results in yeast interaction experiments.
