# Supplementary material for: Yeast–Yeast Interactions: Mechanisms, Methodologies and Impact on Composition
Source: Microorganisms. 2020 Apr 20;8(4):600. doi: 10.3390/microorganisms8040600 (PMC7232261; doi:10.3390/microorganisms8040600)
Supplement: Supplementary file 1 [file microorganisms-08-00600-s001.pdf]

Yeast–Yeast Interactions: Mechanisms, Methodologies and Impact on Composition

Fanny Bordet [1,2,†], Alexis Joran[1,†], Géraldine Klein[1], Chloé Roullier-Gall[1], Hervé Alexandre [1,*]

[1] Univ. Bourgogne Franche-Comté, AgroSup Dijon, PAM UMR A 02.102, F-21000 Dijon, France-IUVV Equipe VAlMiS, rue Claude Ladrey, CEDEX BP 27877, 21078 Dijon, France; bordetfanny@gmail.com (FB); alexis.joran@gmail.com (AJ); geraldine.klein@u-bourgogne.fr (GK.); Chloe.Roullier-Gall@u-bourgogne.fr(CRG)
[2] Lallemand SAS, 19, rue des Briquetiers, CEDEX BP 59, 31702 Blagnac, France
* Correspondence: rvalex@u-bourgogne.fr (HA); Tel.: +33-380-396-393
† These authors contributed equally to this work.

Table S1: Diversity of methodologies and results in yeast interaction experiments

Species		*Saccharomyces cerevisiae / Metschnikowia pulcherrima*								
Matrix		Synthetic Medium						Grape Juice / Must (unspecified color)		
		SGJ	SGJ				SGJ	Grape must		
Sugar Initial Concentration (g/L)		200 (Glc 100 Fru 100)	200 (Glc 100 Fru 100)				200 (Glc 100 Fru 100)	231		
YAN / PAN (mg/L)		YAN 410					200	PAN 154 / NH_3 22		
Temperature (°C)		25	25				20	25		
Oxygenation		++	-	+	++	+++	+	+		
Inoculation delay between 2 strains (h)		0	0				0	0		
Ratio Sc/NS		1:1	1:10				1:10	1:1	1:100	1:10000
Sc population	Max	1	3	3	3	3	4	3	2	3
	Dominance		+					2	2	2
	Decrease	-	-	+	+	+	+	-	-	-
NS population	Max	1	1	1	1	1	1	7	1	3
	Decrease	2 or 1	1	3	3	3	-	7	3	3
	Detectable End	No 2 or 4						No 22	No 7	No 7
Fermentation completion							+	+	+	+
Ethanol		=	=	- -	- -	- -	-	- -	- -	- -
Glycerol			++	- -	- -	- -	+	++	++	++
Organic acids	TA							- -	- -	- -
	VA							- -	- -	- -
	LA									
	AA									
Impacting Factors		SPE / NS	OX / SPE				SPE	RAT / SPE		
Interaction Mechanisms		TOX	COMP				COMP / TOX			
Reference		[65]	[29]				[95]	[32]		

Species		*Saccharomyces cerevisiae / Metschnikowia pulcherrima*											
Matrix		**Red Grape Juice / Must**					**White Grape Juice / Must**						
		Shiraz	Merlot	Shiraz	Tempranillo	Pinot Grigio	Sauvignon blanc	Riesling	Sauvignon blanc	Chardonnay	Muscat commercial grape juice	Sauvignon blanc	Riesling
**Sugar Initial Concentration (g/L)**		210	240	280 (Glc 140 Fru 140)	245	236	219 (Glc 110 Fru 109)	237	217	240 (Glc 120 Fru 120)	200 (supp)	221 (Glc 112 Fru 109)	225
**YAN / PAN (mg/L)**		200	190	YAN 170 (supp)	YAN 181	YAN 236	YAN 588	PAN 147	YAN 170 (supp)	260		YAN 378	YAN 250 (supp)
**Temperature (°C)**		22	22	25		22	20	20	15	22	20	20	20
**Oxygenation**		+	++	++	+	+	+	+	+	+	+	+	+
**Inoculation delay between 2 strains (h)**		24 days	0	96	72	48	48	24	168	24 days	72	48	48
**Ratio Sc/NS**		1:1	1:10	1:1	1:1	1:1	1:10	10:1	1:1	1:1	1:5	1:10	1:1
**Sc population**	**Max**							4			5	3	4
	**Dominance**		+	+									
	**Decrease**		-				-	-			-	-	-
**NS population**	**Max**												0
	**Decrease**	+	2	+	+		+	+	+	15	5	4	2
	**Detectable End**			No 7	No 4-5		No 8	No 15	No 12	No 25	Low 13	No 8	4
**Fermentation completion**		+		17		9	8	+	19	40	10	10	+
**Ethanol**		- -	-	=	- -	-	-	- -	=	-		-	=
**Glycerol**		-	++		++	++		++		+		++	++
**Organic acids**	**TA**						- -		+				- -
	**VA**	+		=	- -				++	++			
	**LA**												
	**AA**	- -	=			=	- -	=	++	- -		- -	=
**Impacting Factors**		GRA		SPE	SPE	SPE / NS	SPE	SPE	SPE	GRA	SPE / TEMP / DEL / MED	DEL	DEL
**Interaction Mechanisms**											COMP	COMP	
**Reference**		[155]	[153]	[36]	[37]	[14]	[47]	[38]	[41]	[155]	[55]	[87]	[44]

Species		*Saccharomyces cerevisiae / Starmerella bacillaris*										
Matrix		**Synthetic Medium**	**Grape Juice / Must (unspecified color)**			**White Grape Juice / Must**						
		SGJ	Grape must			Macabeo	Erbaluce / dried grape must		Sauvignon blanc	Sauvignon blanc	Muscat (commercial grape juice)	Chardonnay / Muscat / Riesling / Sauvignon blanc
**Sugar Initial Concentration (g/L)**		200 (Glc 100 Fru 100)	231			180	403 (Glc 210 Fru 193)		219 (Glc 110 Fru 109)	217	200 (supp)	245
**YAN / PAN (mg/L)**		YAN 410	PAN 154 / NH_3 22			YAN 115			YAN 588	YAN 170 (supp)		YAN 180 adj
**Temperature (°C)**		25	25			20	25		20	15	20 / 28	20
**Oxygenation**		++	+			+	+		+	+	+	+
**Inoculation delay between 2 strains (h)**		0	0			0	0	48	24	144	72	48
**Ratio Sc/NS**		1:1	1:1	1:100	1:10000	1:9	1:1		1:10	1:1	1:5	1:1
**Sc population**	**Max**		2	2	10				3		=	
	**Dominance**	-	-	-	-	-	-	-	-		-	=
	**Decrease**		1	3	10	- +						-
**NS population**	**Max**		1	2	2				1			
	**Decrease**	4	7	10	15	+	6	6/12	-	+	-	7
	**Detectable End**	No 5 / Yes	No 15	No 22	No 22	Low 10				No 14		No 14
**Fermentation completion**			+	+	+	10			7	24		14
**Ethanol**			- -	- -	- -		- -	- -	-	=	=	-
**Glycerol**			++	++	++	++	++	++				++
**Organic acids**	**TA**		- -	- -	- -				+			++
	**VA**		- -	- -	- -							
	**LA**											
	**AA**					++	= / - -	= / - -		++		
**Impacting Factors**		SPE / NS	RAT / SPE			SPE	DEL / SC / NS		SPE	SPE	SPE / TEMP / DEL / MED	GRA / TEMP
**Interaction Mechanisms**		TOX				COMP					COMP	
**Reference**		[65]	[32]			[48]	[23]		[47]	[41]	[55]	[79]

Species		*Saccharomyces cerevisiae / Starmerella bacillaris*																
Matrix		**Red Grape Juice / Must**																
		Merlot			Barbera		Shiraz	Barbera		Cabernet sauvignon / Merlot / Pinot noir / Shiraz		Kotsifali - Manilari 3:1				Barbera	Nebbiolo	Pinot Grigio
**Sugar Initial Concentration (g/L)**		240			244		280 (Glc 140 Fru 140)	246		250		220		214		234 (Glu 118 Fru 116)	234 (Glu 115 Fru 120)	236
**YAN / PAN (mg/L)**							YAN 170 (supp)	YAN 180 adj		YAN 180 adj		YAN 240		YAN 71 + suppl		YAN 180 adj	YAN 180 adj	YAN 236
**Temperature (°C)**		24			25		25	25		25		25		25		25	25	22
**Oxygenation**		+			+		++	+	++	+		+		+		+	+	+
**Inoculation delay between 2 strains (h)**		0	24	48	0	48	96	48		24	48	0	30	0	30	48	48	48
**Ratio Sc/NS**		1:5			1:1		1:1	1:1		1:1		1:1				1:1	1:1	1:1
**Sc population**	**Max**																	
	**Dominance**				-	-				= / +	-	+	-			-	-	
	**Decrease**						+	-	-	-	-		-	-	-	10	-	+
**NS population**	**Max**				4													
	**Decrease**					+	+	4	4	4	4/7	3	8	2	2	7	7	+
	**Detectable End**				No 21	Yes 21	No 7	No 14 / 21	No 14 / Yes 21			Low 5		Low 6	Low 7	No 10 / 14	No 9 / Yes 14	Low 5
**Fermentation completion**		+	+	+	-	-	17	+ / -	+ / -	7	7/10	6	13			14	14	14
**Ethanol**		=	- -	- -	- -	- -	=	- -	- -	- -		-	- -	- -	- -	- - / =	= / - -	-
**Glycerol**			++	++	=	++		++	++	++		=	++	++	++	++	++	++
**Organic acids**	**TA**		++	++						++		=	++			++		
	**VA**						++						++		=			
	**LA**																	
	**AA**							++	+++				++	=	=	- -		
**Impacting Factors**		NS / DEL			DEL / NS / SC		SPE	OX / NS / SC		DEL / GRA		DEL				SC		SPE / NS
**Interaction Mechanisms**								COMP				TOX / COMP					Not TOX / Not COMP / CCC	
**Reference**		[75]			[12]		[36]	[67]		[60]		[49]				[69]	[68]	[14]

**Species**		*Saccharomyces cerevisiae / Torulaspora delbrueckii*												
**Matrix**		**Synthetic Medium**												
		SGJ	WYPD		SGJ	SGJ				SGJ				SGJ	SGJ
**Sugar Initial Concentration (g/L)**		210 (Glc)	200 (Glc)		210 (Glc)	220 (Glc 110 Fru 110)				200 (Glc 100 Fru 100)				200 (Glc 100 Fru 100)	200 (Glc 100 Fru 100)
**YAN / PAN (mg/L)**						YAN 324		YAN 176						YAN 410	200
**Temperature (°C)**		25	25		25	20				25				25	20
**Oxygenation**		+	++	+	+	+				-	+	++	+++	++	+
**Inoculation delay between 2 strains (h)**		0	0		0	0	0	48	48	0				0	0
**Ratio Sc/NS**		1:1	1:1		1:1	1:2	1:20	1:2	1:2	1:10				1:1	1:10
**Sc population**	**Max**	2	1	1	2	2	2	4	5	3	3	3	3	1/3	2
	**Dominance**	+	+	+	+										
	**Decrease**	-	-	-	-	-	-	-	-	-	+	+	+	-	-
**NS population**	**Max**	2	1	1	1	2	3	3	2	1	1	1	1	1	2
	**Decrease**	3	-	-	3	3	4	4	2	1	3	3	3	2/3	-
	**Detectable End**	Low 7			Low 6	Low 5								No 6 / Yes	
**Fermentation completion**		10			10	7	5	9							
**Ethanol**						=	=	-	=	=	- -	- -	- -	=	- -
**Glycerol**										++	- -	- -	- -		=
**Organic acids**	**TA**														
	**VA**														
	**LA**														
	**AA**					=	=	- -							
**Impacting Factors**						RAT / DEL				OX / SPE				SPE / NS	SPE
**Interaction Mechanisms**		Not TOX / Not COMP / CCC	Not TOX / Not COMP / Not QS / CCC		COMP	Not CCC / Not COMP / TOX				COMP				TOX	COMP / TOX
**Reference**		[25]	[26]		[27]	[51]				[29]				[65]	[95]

Species		*Saccharomyces cerevisiae / Torulaspora delbrueckii*							
Matrix		**Grape Juice / Must (unspecified color)**			**White Grape Juice / Must**			**Red Grape Juice / Must**	
			Grape must		Sauvignon blanc	Sauvignon blanc	Riesling	Shiraz	Tempranillo
**Sugar Initial Concentration (g/L)**			231		219 (Glc 110 Fru 109)	217	225	280 (Glc 140 Fru 140)	245
**YAN / PAN (mg/L)**			PAN 154 / NH_3 22		YAN 588	YAN 170 (supp)	YAN 250 (supp)	YAN 170 (supp)	YAN 181
**Temperature (°C)**			25		20	15	20	25	
**Oxygenation**			+		+	+	+	++	+
**Inoculation delay between 2 strains (h)**			0		24	120	48	96	72
**Ratio Sc/NS**		1:1	1:100	1:10000	1:10	1:1	1:1	1:1	1:1
**Sc population**	**Max**	3	10	3	3		4		
	**Dominance**	2		15				+	
	**Decrease**	-	-	-	-		15		
**NS population**	**Max**	3	3	3	1		0		
	**Decrease**	3	10	15	-	+	4	+	+
	**Detectable End**	No 15	No 22	No 22		14	No 6	No 7	Yes 6
**Fermentation completion**		+	+	+	7	19	+	17	
**Ethanol**		- -	- -	- -	-	=	=	=	- -
**Glycerol**		- -	- -	- -			++		++
**Organic acids**	**TA**	+	+	+	+	=	- -		
	**VA**	- -	- -	- -	- -	=		=	- -
	**LA**								
	**AA**					-	=		
**Impacting Factors**			RAT / SPE		SPE	SPE	DEL	SPE	SPE
**Interaction Mechanisms**									
**Reference**			[32]		[47]	[41]	[44]	[36]	[37]

Species		*Saccharomyces cerevisiae / Hanseniaspora guilliermondii*									
Matrix		**Synthetic Medium**								**White Grape Juice / Must**	
		SGJ						SGJ	SGJ	Malvasia fina - Arinto	Malvasia fina - Arinto
**Sugar Initial Concentration (g/L)**		200 (Glc 110 Fru 110)					100	220 (Glc 110 Fru 110)	220 (Glc 110 Fru 110)	234	234
**YAN / PAN (mg/L)**										YAN 175 / 387 (supp)	YAN 387
**Temperature (°C)**		18						20	25	20	20
**Oxygenation**		+						+	+	+	+
**Inoculation delay between 2 strains (h)**		0						0	0	0	0
**Ratio Sc/NS**		1:1	3:1	1:2	1:10	1:100	1:10	1:1	1:1	1:1	1:1
**Sc population**	**Max**	2	3	7	7	7			2	1	1
	**Dominance**										-
	**Decrease**	-	-	-	-	-		-	-	-	-
**NS population**	**Max**	1			3	3			2	1	1
	**Decrease**	1	+	1	4	15	7	+	2	2	6
	**Detectable End**	No 7	No 3	No 8	No 11	No 23	Yes 34	Low 4	No 4	No 12	Low 10
**Fermentation completion**		20	20	25	25	25		+	9	+	
**Ethanol**										-	
**Glycerol**											
**Organic acids**	**TA**									=	
	**VA**									=	
	**LA**									=	
	**AA**									=	
**Impacting Factors**		RAT								MED	
**Interaction Mechanisms**		TOX / Not COMP						TOX		Not COMP	
**Reference**		[52]						[85]	[99]	[50]	[112]

Species		*Saccharomyces cerevisiae / Hanseniaspora uvarum*			
**Matrix**		**Synthetic Medium**			**White Grape Juice / Must**
		SGJ	SGJ	SGJ	Macabeo
**Sugar Initial Concentration (g/L)**		200 (Glc 110 Fru 110)	200 (Glc 100 Fru 100)	200 (Glc 100 Fru 100)	180
**YAN / PAN (mg/L)**			YAN 410	YAN 200	YAN 114.6
**Temperature (°C)**		18	25	20	20
**Oxygenation**		+	++	+	+
**Inoculation delay between 2 strains (h)**		0	0	0	0
**Ratio Sc/NS**		4:1	1:1	1:10	1:9
**Sc population**	**Max**	2		1	
	**Dominance**				
	**Decrease**	-		8	
**NS population**	**Max**		1	1	1
	**Decrease**	1	2	1	5
	**Detectable End**	No 3	No 4	Low 10	No 10
**Fermentation completion**		18			+
**Ethanol**			=	+	=
**Glycerol**				-	++
**Organic acids**	**TA**				
	**VA**				
	**LA**				
	**AA**				++
**Impacting Factors**		RAT	SPE / NS	SPE	SPE
**Interaction Mechanisms**		TOX / Not COMP	TOX	COMP / TOX	COMP
**Reference**		[52]	[65]	[95]	[48]

YAN = yeast assimilable nitrogen / **PAN** = primary amino nitrogen. **Sc** = *S. cerevisiae* / **NS** = Non-*Saccharomyces.* **TA** = total acidity / **VA** = volatile acidity / **LA** = lactic acid / **AA** = acetic acid. **SGJ** = Synthetic Grape Juice / **Glc** = Glucose / **Fru** = Fructose / **WYPD** = Yeast Peptone Dextrose medium modified for wine fermentation / **Supp** = with supplementation. **Oxygen**: **-** = anaerobia, +/- semi-anaerobia, low oxygenation, + = semi-anaerobia, ++ aerobia, +++ aerobia, with higher oxygenation. **Population columns**: **Max =** maximal population reached by day x / **Dominance**: + = dominance of *S. cerevisiae*, = similar populations, "x" = dominance obtained after x days / **Decrease** = decrease since day x / **Low** = low population since day x / **No** = population not detectable since day x / **Yes** = population still detectable at day x. **Fermentation completion**: x = reached at day x, +/- = reached/not reached during experimentation. **Ethanol, glycerol and organic acids** are compared to Sc pure culture: +++ very high increase ++ high increase, + increase, +/- slight decrease, = no change, - decrease, - - high decrease. **Impacting factors**: inoculation delay (**DEL**), inoculation ratio between *S. cerevisiae* and non-*Saccharomyces* yeast (**RAT**), yeast species (**SPE**), yeast strain *S. cerevisiae* (**SC**) or non-

Saccharomyces (**NS**), medium composition (**MED**), grape nature (**GRA**), temperature (**TEMP**), oxygenation (**OX**), type of reactor (lab, pilot, industrial) (**REAC**). **Interaction mechanisms**: involvement of quorum sensing mechanisms (**QS**), toxic compounds (including ethanol, antimicrobial peptides) (**TOX**), competition for nutrient (including oxygen) (**COMP**), cell-cell contact mechanisms (**CCC**) / **No** = mechanism involvement has been ruled out by the study.
